# Sexually antagonistic coevolution of the male nuptial gift and female feeding behaviour in decorated crickets

**DOI:** 10.1098/rspb.2024.0804

**Published:** 2024-07-03

**Authors:** Samuel Burns-Dunn, Tassie Mortys, Clarissa M. House, Christopher Mitchell, Kristin R. Duffield, Bert Foquet, Ben M. Sadd, Scott K. Sakaluk, John Hunt

**Affiliations:** ^1^ School of Science, Western Sydney University, Hawkesbury Campus, Richmond, NSW 2753, Australia; ^2^ Centre for Ecology & Conservation, School of Biosciences, University of Exeter, Cornwall Campus, Penryn TR10 9EZ, UK; ^3^ Crop BioProtection Research Unit, Agricultural Research Services, United States Department of Agriculture, National Centre for Agricultural Utilization Research, Peoria, IL, USA; ^4^ School of Biological Sciences, Illinois State University, Normal, IL 61790-4120, USA

**Keywords:** interlocus sexual conflict, nuptial gift, spermatophylax, free amino acids, experimental evolution

## Abstract

The evolution of nuptial gifts has traditionally been considered a harmonious affair, providing benefits to both mating partners. There is growing evidence, however, that receiving a nuptial gift can be actively detrimental to the female. In decorated crickets (*Gryllodes sigillatus*), males produce a gelatinous spermatophylax that enhances sperm transfer but provides little nutritional benefit and hinders female post-copulatory mate choice. Here, we examine the sexually antagonistic coevolution of the spermatophylax and the female feeding response to this gift in *G. sigillatus* maintained in experimental populations with either a male-biased or female-biased adult sex ratio. After 25 generations, males evolving in male-biased populations produced heavier spermatophylaxes with a more manipulative combination of free amino acids than those evolving in female-biased populations. Moreover, when the spermatophylax originated from the same selection regime, females evolving in male-biased populations always had shorter feeding durations than those evolving in female-biased populations, indicating the evolution of greater resistance. Across populations, female feeding duration increased with the mass and manipulative combination of free amino acids in the spermatophylax, suggesting sexually antagonistic coevolution. Collectively, our work demonstrates a key role for interlocus sexual conflict and sexually antagonistic coevolution in the mating system of *G. sigillatus*.

## 1. Introduction

While sexual reproduction requires at least some degree of cooperation between males and females, the reproductive interests of the sexes are almost never perfectly aligned [1]. This occurs because the sexes have divergent reproductive optima, in which traits favoured by one sex are often costly to the other [2]. Whenever this occurs, there will inevitably be conflict over the outcome of reproductive interactions—a process known as interlocus sexual conflict [2,3]. Males are expected to evolve sexually antagonistic adaptations to manipulate females, and females to evolve counter-adaptations to prevent this manipulation from biasing the outcome of these interactions towards their own reproductive interests [2,3]. The result is a perpetual cycle of sexually antagonistic coevolution between the interacting traits in males and females [2] that can drive rapid trait evolution (e.g. [4–6]) and facilitate population divergence (e.g. [7–9]). Under certain conditions, theory predicts that this evolutionary arms race can also promote speciation (e.g. [10–13]) and even increase the risk of population extinction (e.g. [14–16]).

In many species, the traits mediating interlocus sexual conflict are well known and often extravagant. For example, male bed bugs (*Cimex lectularius*) use a modified paramere to pierce the female’s abdomen and inseminate directly into the body cavity, ensuring last-male sperm precedence but reducing female lifespan and reproductive success [17–20]. In other species, the traits mediating interlocus sexual conflict are far more subtle, involving chemicals transferred during mating. A classic example is the cocktail of seminal fluid proteins contained in the ejaculate of male *Drosophila melanogaster* that boosts short-term egg laying [21] but reduces female lifespan [22,23]. While this form of chemical manipulation is likely to be common, we still know relatively little about the role of chemicals in interlocus sexual conflict in systems other than *D. melanogaster*.

Nuptial gifts are materials beyond the obligatory gametes provided by a donor to a recipient during courtship or copulation to enhance donor fitness [24]. In most sexually reproducing animals, gift donors are typically male and recipients are female [24], although reversal of these typical roles does exist (e.g. [25]). Nuptial gifts are taxonomically widespread in insects and come in a large diversity of forms [26–29] that can be captured in two broad dimensions [24,26]. The first dimension classifies nuptial gifts according to their origin, including those manufactured or sequestered by the donor (endogenous gifts) or food items that are captured or collected by the donor (exogenous gifts) [24,26]. The second dimension classifies how the gift is received by the recipient, either absorbed through the digestive system (oral gifts) or the reproductive tract (genital gifts) or injected directly through the recipient’s body wall (transdermal gifts) [24,26]. There has been considerable debate over how this diversity in nuptial gifts has evolved [26–29]. Initial arguments treated the evolution of nuptial gifts as a harmonious affair, with the giving and receiving of a nuptial gift being mutually beneficial to both sexes [24,26–28]. While many examples exist showing that nuptial gifts benefit both sexes, there is growing evidence that this outcome is far from universal [26–28]. Indeed, for many insect species, receiving a nuptial gift can be actively detrimental to the female by reducing her lifespan (e.g. [30,31]), increasing the allocation of resources to immediate gamete production (e.g. [32,33]), inducing longer than optimal refractory periods (e.g. [33,34]) or reducing the optimal degree of polyandry (e.g. [35,36]). While these examples implicate an important role for interlocus sexual conflict in the evolution of nuptial gifts, there is surprisingly little direct empirical support for this process, especially for the sexually antagonistic coevolution that is predicted to occur between the male nuptial gift and the female response to this gift (but see [37–39]).

In the decorated cricket (*Gryllodes sigillatus*), males produce an externally attached spermatophore that is transferred to the female at mating. The spermatophore consists of a sperm-containing capsule (the ampulla) and a much larger, gelatinous nuptial gift (the spermatophylax). Immediately after the spermatophore has been transferred, the female dismounts the male, detaches the spermatophylax from the ampulla with her mandibles and starts feeding on it. While feeding on the nuptial gift, sperm are evacuated into her reproductive tract from the ampulla. After the spermatophylax is fully consumed or prematurely discarded, the female immediately removes and consumes the ampulla, terminating sperm transfer. Females vary considerably in the length of time they spend feeding on the spermatophylax and the longer the female is delayed from removing the ampulla, the more sperm is transferred to her sperm storage organ [35,40,41]. Sperm transfer follows a diminishing returns function, with complete sperm transfer occurring after the ampulla has been attached for 50 min [35]. The size [40] and chemical composition [42] of the spermatophylax significantly influence female feeding behaviour and prolong ampulla attachment time in *G. sigillatus*. Females take longer to fully consume a large versus a small spermatophylax [40]. The spermatophylax consists mostly of water (~80%), with the remaining dry mass consisting of spermatophylax proteins [43] and a mixture of 19 free amino acids [42,44]. Many of these free amino acids are phagostimulants in insects [45], which explains the readiness of females to accept the spermatophylax in *G. sigillatus*, as well as other non-gift giving cricket species [46,47]. The free amino acid composition also influences the amount of time that a female *G. sigillatus* spends feeding on the spermatophylax [42,44]. Using a multivariate selection analysis, Gershman *et al*. [42] showed that a specific combination of free amino acids decreased the probability that a female would prematurely discard a spermatophylax, most likely by altering the texture or gustatory appeal of this nuptial gift. As females are highly polyandrous and the outcome of sperm competition conforms to a numerical lottery in this species [48,49], the length of time that a female spends feeding on the spermatophylax is a major determinant of male paternity [50,51]. Thus, even though spermatophylax production reduces the immune function of the male [52,53], consumption of this nuptial gift by the female clearly enhances male fitness.

In contrast to males, females appear to receive little direct nutritional benefit from consuming a spermatophylax [54–56]. In an experiment that simultaneously manipulated the number of spermatophylaxes that females were permitted to consume each day and the total amount of food available to these females, there was no effect of spermatophylax consumption on female survival, egg size or lifetime reproductive output, even when females were completely deprived of food [54]. This result has been replicated in our laboratory [56], as well as by others [55]. Rather, consuming a spermatophylax is costly to a female as it causes her to relinquish some of her control over the paternity of her offspring. Female *G. sigillatus* express strong post-copulatory mate choice by removing the ampulla of unattractive males sooner [57] and in approximately 25% of matings, the female prematurely discards the spermatophylax before sperm transfer is complete [35,41]. Females also benefit from polyandry (after controlling for the number of gifts consumed) by producing more offspring that survive to reproductive maturity than monogamous females [56]. As both processes will be disrupted when consuming a gift for longer, it will cause the female to relinquish her control over the paternity of her offspring. In addition, there are also compounds (most likely sex proteins [43]), in the spermatophyax that decrease female sexual receptivity in *G. sigillatus* [47,58], which is likely to further increase these costs to females. Consequently, it has been argued that interlocus sexual conflict has played a key role in the evolution of nuptial gifts in *G. sigillatus* [42,46,47,59]. Importantly, there is a positive genetic correlation between the combination of free amino acids in the spermatophylax that prolongs female feeding and female spermatophylax feeding duration, which indicates that the genes expressed in males to produce more manipulative spermatophylaxes are positively linked to genes expressed in females that make them more vulnerable to being manipulated (i.e. lower resistance) [59]. While this finding is consistent with an evolutionary history of sexually antagonistic coevolution over the consumption of this nuptial gift in *G. sigillatus* [59], we currently do not have any direct empirical support for this process.

In this study, we examine the sexually antagonistic coevolution of the male nuptial gift (the spermatophylax) and the female feeding response to this gift in *G. sigillatus*. To achieve this, we use an experimental evolution approach in which we exposed replicated populations of crickets to an elevated or reduced intensity of interlocus sexual conflict by manipulating the adult sex ratio to be either male-biased or female-biased, respectively. After 25 generations, we quantified differences in the dry mass and free amino acid composition of the spermatophylax collected from males in each population. Next, we used a reciprocal crossing design within each pair of replicate populations to quantify the feeding behaviour of females evolving in a male- or female-biased population when receiving a spermatophylax from either a male evolving in a male- or female-biased population. If interlocus sexual conflict is an important process in the evolution of nuptial gifts, we predict that males evolving in male-biased populations will produce spermatophylaxes that are heavier and contain a more manipulative combination of free amino acids compared with males evolving in female-biased populations. Furthermore, we predict that when the spermatophylax originates from the same selection regime, females evolving in male-biased populations will evolve shorter feeding durations on this gift than those evolving in female-biased populations due to the evolution of greater resistance in these females. Finally, if interlocus sexual conflict generates sexually antagonistic coevolution, we predict a positive relationship between female feeding duration and both the mass and manipulative free amino composition of the spermatophylax across populations. As females with shorter spermatophylax feeding times are more resistant in *G. sigillatus*, this equates to a negative relationship between male manipulation and female resistance.

## 2. Material and methods

### (a) Experimental evolution procedure

*Gryllodes sigillatus* used in this study were taken from a mass colony that is the descendants of approximately 500 adult crickets collected from Las Cruces, New Mexico, in 2001. The mass colony is distributed across 12 transparent 15 l plastic containers and housed in an environmental chamber (Percival I−66VL) maintained at 32 ± 1°C with a 14L : 10D light cycle. Crickets were provided ad libitum with a 50–50% mixture of commercial cat (Friskies 7; Nestle Purina PetCare, Australia) and rat (Specialty Feeds, Australia) pellets, water in 60 ml glass test tubes plugged with cotton wool and cardboard egg cartons for shelter. Food and water were replenished weekly. When adults were detected, a 10 cm Petri dish containing moistened cotton wool was added to the container as an oviposition substrate. Hatching nymphs were collected en masse, and approximately 500 nymphs were allocated at random to each 15 l container to establish the next generation. This process ensures gene flow in each generation to promote the maintenance of genetic variation in the mass colony.

To experimentally alter the intensity of interlocus sexual conflict, we manipulated the adult sex ratio (male or female-biased) in paired replicate populations. To establish the first pair of replicate populations, we collected approximately 4000 newly hatched nymphs from the mass colony. Half of these nymphs were allocated at random to a 110 l plastic container to serve as the starting generation for the male-biased population and the remaining half to a second 110 l container to serve as the starting generation for the female-biased population. Each container was provided ad libitum with food and water, as well as an abundance of cardboard egg cartons for shelter as described above for the mass colony. This procedure was repeated each day for six consecutive days (in June 2018) to produce six paired replicates of each selection regime (i.e. one paired replicate established per day) (electronic supplementary material, figure S1). Populations were maintained in a constant temperature room set to the same temperature and lighting conditions as the mass colony, with food and water being replenished weekly.

When fourth instar nymphs were detected in a population, the population was sorted into single-sex containers. For each population, 500 nymphs of each sex were established at random in separate 40 l containers and provided with food, water and shelter as outlined above. These single-sex populations were checked daily and when most crickets in both populations were adults, we selected crickets at random from these populations to establish a new population where we manipulated the adult sex ratio. The male-biased population consisted of 250 adult males and 50 adult females, whereas the female-biased population consisted of 50 adult males and 250 adult females (electronic supplementary material, figure S1). Each population was placed in a separate 110 l container and provided ad libitum with food and water, plus an abundance of cardboard egg cartons. We also provided each population with eight 9 cm diameter Petri dishes filled with moist cotton wool for oviposition. These oviposition pads were watered every 2 days for a total of 10 days and then established in individual containers (10 × 10 × 5 cm) lined with paper towels. When eggs hatched, nymphs were collected and approximately 2000 were selected at random from across the eight containers per population to establish the next generation in a 110 l container (as described above). The adult population was then killed by placing the container at −20°C for 3 h. Selection regimes were maintained for a total of 25 generations.

In generation 26, adults in all populations were maintained at the same population size as in previous generations but with an equal sex ratio (i.e. 150 crickets of each sex) (electronic supplementary material, figure S1). Adults were maintained, and oviposition was collected following the protocols outlined for previous generations. When eggs hatched, we established 300 newly hatched nymphs at random from each population (total *n* = 3600 nymphs) in individual containers (5 × 5 × 5 cm) to remove any common environment effects on male spermatophylax properties and female feeding behaviour. Each container was provided with a pellet of cat food, water in a 3.5 ml test tube plugged with a cotton ball and a piece of egg carton for shelter. Fresh food and water were provided, and containers were cleaned each week. At the final instar, nymphs were checked daily for eclosion to adulthood.

### (b) Spermatophylax sampling and analysis

At 5 days post-eclosion, we collected a single spermatophylax from 80 adult males per population (total *n* = 960 spermatophylaxes). Of these, 20 spermatophylaxes per population (total *n* = 240 spermatophylaxes) were selected at random to measure dry mass and free amino acid composition. An additional 48 spermatophylaxes per population (total *n* = 576 spermatophylaxes) were used to assess female spermatophylax feeding behaviour (electronic supplementary material, figure S1). The remaining 12 spermatophylaxes per population (total *n* = 144 spermatophylaxes) were stored at −80°C as a reserve.

We collected a spermatophylax from each male by gently squeezing the spermatophore pouch, causing him to extrude the spermatophylax. The spermatophylax was then detached from the spermatophore pouch using a pair of fine forceps and immediately sealed in an airtight microcentrifuge vial and stored at −80°C until use. Forceps were washed in 80% ethanol between collecting successive spermatophylaxes.

Each of the 20 spermatophylaxes per population used to quantify dry weight and free amino acid composition was freeze-dried for 24 h using a Labconco Freeze-drier (Labconco, Kansas City, MO, USA) and then weighed using an electronic balance (Ohaus Explorer Professional model EP214C, NJ, USA). We quantified the free amino acid composition of each spermatophylax using an EZ:faast reagent kit (Phenomenex, Torrance, CA, USA) optimized for gas chromatography-mass spectrometry, following the procedure outlined in Gershman *et al*. [42]. Full details of this procedure are outlined in Text S1.

### (c) Analysis of female spermatophylax feeding behaviour

At 5 days post-eclosion, we assessed the spermatophylax feeding behaviour of 48 females per population (total *n* = 576 females). For each pair of replicate populations experiencing contrasting selection regimes, half of the females (total *n* = 288 females) were selected at random to receive a spermatophylax originating from the male-biased population, while the remaining half (total *n* = 288 females) were to receive a spermatophylax originating from the female-biased population (electronic supplementary material, figure S1).

Female spermatophylax feeding behaviour was observed in small plexiglass arenas (10 × 4 × 7.5 cm) during the dark phase of the light cycle under red light illumination at 30°C. One female was introduced into each arena and allowed to acclimate for 3 min. A single spermatophylax from the appropriate treatment that had been thawed to room temperature was presented to each female on the tip of a blunt dissecting probe. The spermatophylax was offered to the female by touching the spermatophylax to the female’s palps. This process was repeated until the female either accepted the spermatophylax (i.e. took hold with her mouth parts and started feeding) or withdrew from it. If the female withdrew after multiple attempts, the spermatophylax was gently placed on the floor of the arena directly in front of the female’s palps. If the female did not accept the spermatophylax in 20 min, both the female and spermatophylax were excluded from the experiment. For females that accepted the spermatophylax, we observed the female for 50 min and recorded the amount of time spent feeding on the spermatophylax. We used 50 min as the observation period as this is the time required for complete sperm transfer in *G. sigillatus* [35].

### (d) Statistical analysis

We examined the effect of the selection regime experienced by the male on spermatophylax mass using a linear mixed model (LMM) that included the selection regime as a fixed effect, replicate population × selection regime as a random effect and dry spermatophylax weight for each male as the response variable (electronic supplementary material, figure S1). In this model, we used a Restricted Maximum Likelihood (REML) estimation, Satterthwaite’s approximation for the degrees of freedom, and 10 000 bootstraps of the data to estimate the 95% confidence intervals (CIs) for the variance explained by the random effect.

We examined the effect of the selection regime experienced by the male on the free amino acid composition of the spermatophylax in two ways. First, due to the large number of free amino acids contained in the spermatophylax, we used Discriminant Function Analysis (DFA) to determine if the replicate populations maintained on the different selection regimes could be accurately classified according to the free amino acid composition of the spermatophylax. Prior to analysis, we log_10_ transformed the quantity of each free amino acid in the spermatophylax to ensure normality. We performed the analysis on 12 groups, with each group representing a different replicate population in each selection regime. Discriminant functions (DFs) with eigenvalues exceeding one were retained for further analysis and we considered factor loadings that are |0.25| or greater to be biologically important [60]. Wilks’s lambda values, which were converted to a chi-square value for significance testing, were used to examine whether the resulting DFs significantly explained differences in free amino acids between our groups. We used a ‘leave-one-out’ cross-validation to determine the percentage of spermatophylaxes correctly classified into their original groups.

Second, we examined the specific combination of free amino acids that influences female feeding behaviour in *G. sigillatus* and determined whether this differed across our selection regimes. Previously, a multivariate selection analysis [42] was used to show that the combination of free amino acids that stimulates the female to continue feeding on a spermatophylax (w) can be described by the vector of linear selection gradients,


(2.1) $$w=\left(-0.034PC_{1}\right)+\left(-0.177PC_{2}\right)+\left(-0.181PC_{3}\right),$$

where PC1, PC2 and PC3 represent the three PCs that describe the variation in free amino acid composition in Gershman *et al*. [42]. Using this equation, we calculated a multivariate ‘manipulation’ score for each spermatophylax following the protocol outlined in the elctronic supplementary material, text S2. This produced a manipulation score for each spermatophylax in the experiment, whereby larger values mean that the free amino acid composition of the spermatophylax is more manipulative (less likely to be prematurely discarded by the female) and smaller values represent spermatophylaxes that are less manipulative (more likely to be prematurely discarded). Importantly, the crickets used in the work of Gershman *et al*. [42] and to establish our experimental evolution populations were taken from the same mass colony. Moreover, equation (2.1) has been used with success to document the genetic correlation between the manipulative combination of free amino acids in the spermatophylax and female feeding time [59] and how the manipulative combination of free amino acids changes during terminal investment [61], indicating it has predictive power in this mass colony. We examined the effect of the selection regime experienced by the male on these spermatophylax manipulation scores using an LMM that included the selection regime as a fixed effect, replicate population × selection regime as a random effect and the manipulation score for each spermatophylax as the response variable (electronic supplementary material, figure S1). We used REML estimation, Satterthwaite’s approximation for the degrees of freedom, and 10 000 bootstraps of the data to estimate the 95% CIs for the variance explained by the random effect.

We examined the effect of the selection regime experienced by the female, the selection regime experienced by the male donating the spermatophylax and their interaction on the spermatophylax feeding duration of females using an LMM. In this model, we included the selection regime of the female receiving the spermatophylax, the selection regime of the male donating the spermatophylax and their interaction as fixed effects, replicate population nested within the interaction between female and male selection regime as a random effect and the female spermatophylax feeding duration of each female as the response variable (electronic supplementary material, figure S1). As with the analysis of spermatophylax properties, we used REML estimation, Satterthwaite’s approximation for the degrees of freedom, and 10 000 bootstraps of the data to estimate the 95% CIs for the variance explained by the random effect. Prior to this analysis, we excluded from the data set any female that did not feed on the spermatophylax for at least 2 min, as we could not be certain whether the female accidentally dropped or actively discarded the spermatophylax. This occurred for 35 of the 576 (6.08%) females observed. The number of females we excluded from each replicate population did not differ across the selection regime experienced by the female (*F*_1,20_ = 0.03, *p* = 0.86) or the selection regime experienced by the male donating the spermatophylax (*F*_1,20_ = 0.29, *p* = 0.59), nor was there an interaction between these main effects (*F*_1,20_ = 0.02, *p* = 0.85).

We examined the relationship between mean spermatophylax properties (mass and the free amino acid manipulation score) and mean female spermatophylax feeding duration across populations using a regression analysis. In both analyses, the respective mean spermatophylax property was included as the predictor variable and the mean feeding duration of females when given a spermatophylax from a male experiencing the same selection regime was included as the response variable.

All statistical analyses were conducted in IBM SPSS Statistics (v. 29.0.0.0).

## 3. Results

**Figure 1.**
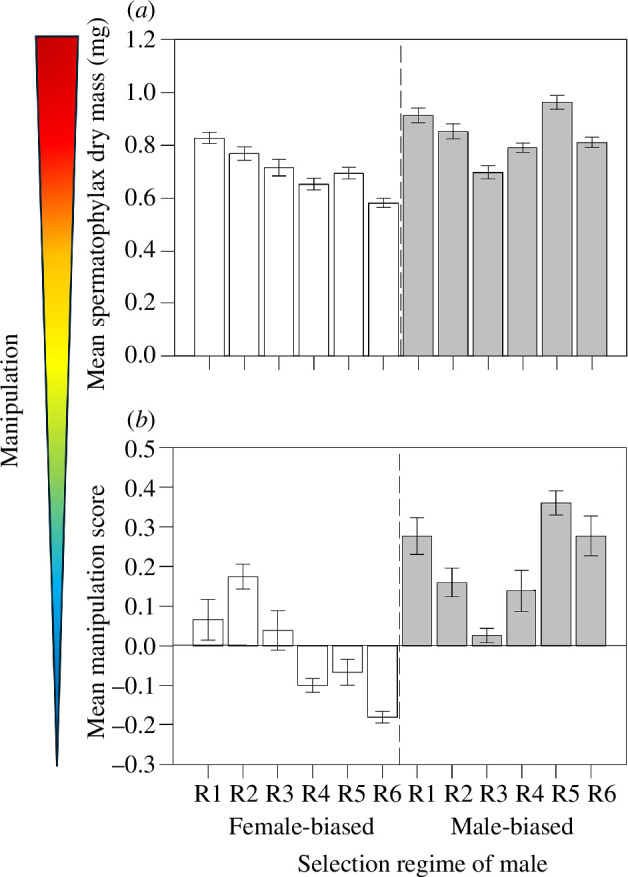
Mean (± s.e.) spermatophylax dry mass (*a*) and multivariate manipulation scores (*b*) across the six replicate populations (R1 to R6) experiencing divergent selection regimes (female or male-biased). White bars represent spermatophylaxes taken from males evolving in female-biased populations, whereas grey bars represent spermatophylaxes taken from males evolving in male-biased populations. The heat arrow represents the degree of male manipulation, with red representing increased manipulation and blue representing decreased manipulation.

There was a significant effect of the selection regime experienced by a male (*F*_1,10.01_ = 6.31, *p* = 0.031) on spermatophylax mass, with males from male-biased populations producing heavier spermatophylaxes than those from female-biased populations (figure 1*a*). There was also considerable variation in spermatophylax mass across our replicate populations (variance = 0.008, 95% CIs: 0.006, 0.011), with some replicate populations producing heavier spermatophylaxes (e.g. replicate 1) than others (replicate 3) (figure 1*a*).

**Figure 2.**
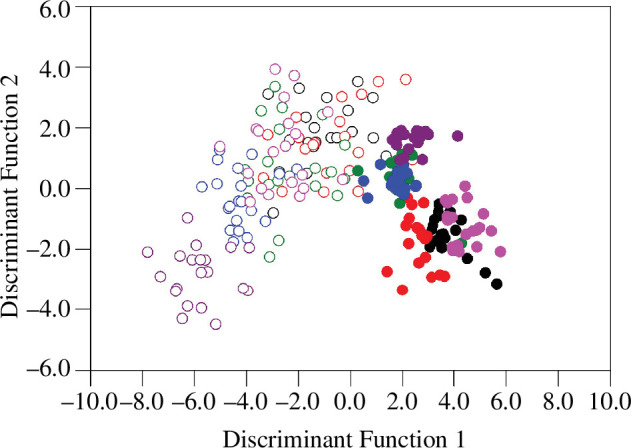
Discriminant function 1 and 2 scores for the 12 groups (each replicate per selection regime) were used in the analysis of the free amino acid composition of the spermatophylax. Open symbols represent spermatophylaxes from the female-biased populations, whereas closed symbols represent those from the male-biased populations. The replicate populations in each selection regime are colour-coded: black = replicate 1, red = replicate 2, green = replicate 3, blue = replicate 4, pink = replicate 5 and purple = replicate 6.

DFA revealed two eigenvectors with eigenvalues exceeding one, which collectively explained 81.42% of the variation in free amino acids in the male spermatophylax. DF1 explained 66.77% of this variation and was negatively loaded to three free amino acids (hydroxyproline, phenylalanine and lysine) (table 1). DF1 significantly discriminated across groupings (table 1), with a clear separation between male-biased and female-biased populations (figure 2). Spermatophylaxes produced by males from male-biased populations had higher DF1 scores than those produced by males from female-biased populations, meaning they had lower amounts of hydroxyproline, phenylalanine and lysine (table 1, figure 2). DF2 explained the remaining 14.65% of this variation and was positively loaded with five free amino acids (leucine, threonine, asparagine, methionine and tryptophan) (table 1). DF2 also significantly discriminated across grouping (table 1), but this separation was predominantly between replicate populations rather than the selection regime (figure 2). However, there were no consistent patterns in how DF2 varied across replicate populations (figure 2). These DFs performed well in distinguishing between groups based on the free amino acids in the spermatophylax, with a total of 71.30% of cross-validated group cases being correctly classified.

**Table 1.** Discriminant function analysis of the free amino acid composition of the spermatophylax from each replicate per selection regime (12 groups in total). We show the first two discriminant functions that have eigenvalues exceeding one, and we interpret factor loadings of |0.25| or greater as biologically important (in bold). Wilks’s lambda values (which are converted to a chi-square value for significance testing) are provided to determine whether each discriminant function explains differences in free amino acids between our groups.

	**discriminant functions**	
	1	2
eigenvalue	10.51	2.31
% variance	66.77	14.65
canonical correlation	0.96	0.84
Wilks’s lambda	0.003	0.029
*χ* ^2^	1334.51	788.48
d.f.	209	180
*p-*value	0.0001	0.0001
**free amino acids**		
alanine	−0.05	0.20
glycine	−0.03	0.11
valine	−0.12	0.04
leucine	−0.12	**0.26**
isoleucine	−0.07	0.04
threonine	−0.04	**0.27**
serine	−0.01	0.05
proline	−0.05	0.10
asparagine	0.01	**0.27**
aspartic Acid	0.02	0.17
methionine	0.04	**0.30**
hydroxyproline	**−0.37**	0.09
glutamine	−0.05	0.13
phenylalanine	**−0.32**	0.14
glutamine	−0.14	0.02
lysine	**−0.25**	0.19
histidine	−0.19	0.09
tyrosine	−0.01	0.16
tryptophan	0.04	**0.27**

Most importantly, the selection regimes experienced by the male significantly influenced the multivariate manipulation score of the spermatophylax (*F*_1,10.01_ = 9.15, *p* = 0.013), with males from male-biased populations producing a more manipulative combination of free amino acids in the spermatophylax than those from female-biased populations (figure 1*b*). However, there was also considerable variation in these manipulation scores across our replicate populations (variance = 0.014, 95% CIs: 0.011, 0.021), with some replicate populations having higher scores (e.g. replicate 5) than others (e.g. replicate 3) (figure 1*b*).

**Figure 3.**
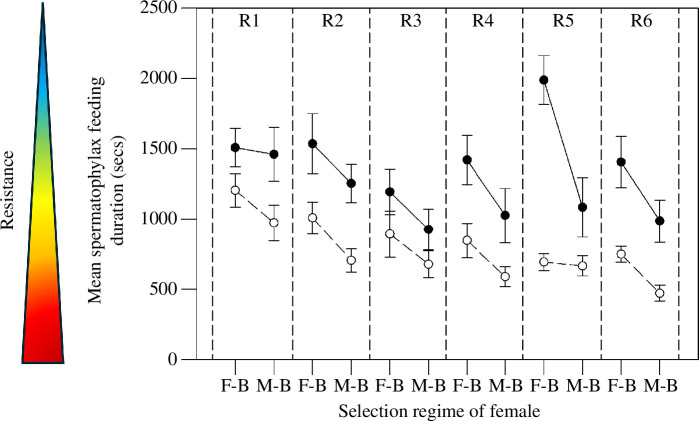
The mean (±s.e.) spermatophylax feeding duration of females from each of the six replicate populations (R1 to R6) per selection regime when given a spermatophylax from a male evolving in a female-biased (open symbols, dashed lines) or a male-biased population (closed symbols, dashed lines) from the same pair of replicate populations. The heat arrow represents the degree of female resistance, with red representing increased resistance and blue representing decreased resistance.

There was a significant effect of the selection regime experienced by the female on spermatophylax feeding duration (*F*_1,19.79_ = 26.11, *p* = 0.002), with females evolving in male-biased populations having, on average, shorter spermatophylax feeding durations than those evolving in female-biased populations (figure 3). There was also a significant effect of the selection regime experienced by the male donating the spermatophylax on female feeding duration (*F*_1,19.79_ = 38.30, *p* = 0.0001), with females receiving a spermatophylax from males evolving in male-biased populations having, on average, longer feeding durations than those receiving a spermatophylax from males evolving in female-biased populations (figure 3). The interaction between these main effects, however, was not significant (*F*_1,19.79_ = 0.91, *p* = 0.35). Female spermatophylax feeding durations also varied substantially across replicate populations (variance = 22517.31, 95% CIs: 15533.42, 77206.38), with feeding durations being longer in some replicate populations (e.g. replicate 5) than others (e.g. replicate 3) (figure 3).

**Figure 4.**
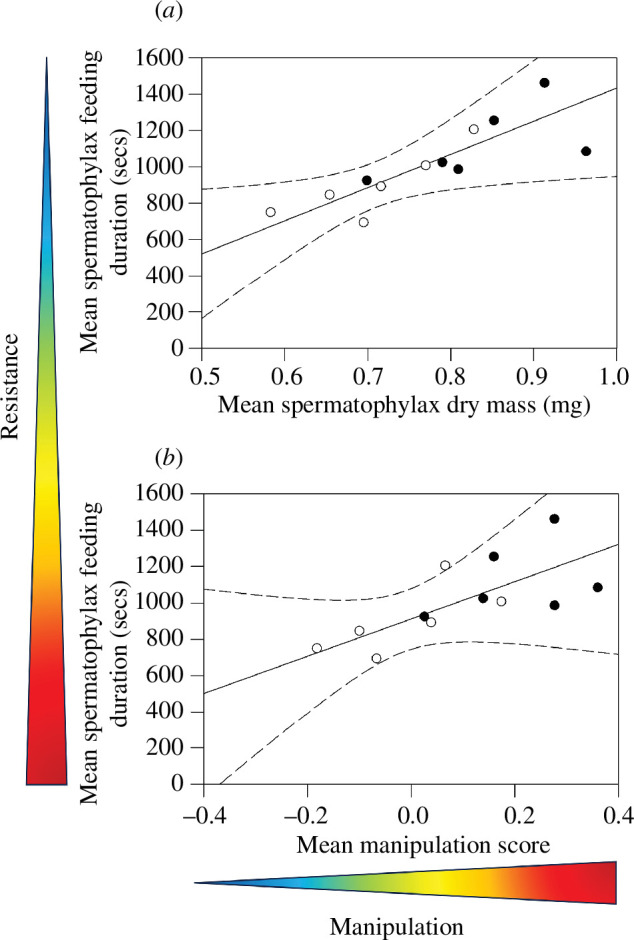
The relationship between mean dry spermatophylax mass (*a*), mean spermatophylax manipulation score (*b*) and mean female spermatophylax feeding duration across populations when the females are given a spermatophylax from their own population. In each figure, closed symbols represent crickets from the male-biased populations, and open symbols represent crickets from the female-biased population. In each figure, the solid line represents the least squares regression line, and the dashed lines represent the 95% confidence intervals for this regression line. The heat arrow represents the degree of male manipulation and female resistance, with red representing increased manipulation and resistance and blue representing decreased manipulation and resistance.

Female spermatophylax feeding duration increased (reduced resistance) with both spermatophylax dry mass (figure 4*a*; adjusted *R*^2^ = 0.63, *F*_1,11_ = 19.40, *p* = 0.001) and manipulation score (figure 4*b*; adjusted *R*^2^ = 0.43; *F*_1,11_ = 9.42, *p* = 0.012) across populations.

## 4. Discussion

Nuptial gifts are taxonomically widespread and encompass a striking diversity of forms across the animal kingdom, especially in insects [26–29]. Initial explanations for the evolution of nuptial gifts centred on the idea that both sexes benefit by giving or receiving a gift [24,26–28]. Several recent studies, however, have shown that receiving a nuptial gift can be detrimental to the female (e.g. [26–28]). While this suggests an important role for intersexual sexual conflict in the evolution of nuptial gifts, there is surprisingly little direct empirical evidence for species other than *D. melanogaster*. Here, we used experimental evolution to test the importance of interlocus sexual conflict directly on the evolution of the male nuptial gift and female feeding behaviour in response to this gift in *G. sigillatus*. After 25 generations of evolution in response to elevated (male-biased) or reduced (female-biased) interlocus sexual conflict, we show that there is significant divergence in both the manipulative properties of the spermatophylax and female feeding behaviour across selection regimes. Males evolving in male-biased populations were shown to produce heavier spermatophylaxes with a more manipulative combination of free amino acids than those from female-biased populations. Furthermore, when the spermatophylax originated from the same selection regime, females evolving in male-biased populations always had shorter feeding durations than those evolving in female-biased populations indicating the evolution of greater resistance in these females. Finally, we show that female feeding duration increased with both the mass and manipulative combination of free amino acids in the spermatophylax across populations, indicating the sexually antagonistic coevolution of these traits. Our work, therefore, demonstrates a clear and important role for interlocus sexual conflict in the coevolution of male nuptial gifts and female responses to these gifts in *G. sigillatus*.

Our finding that males produce heavier spermatophylaxes with a more manipulative combination of free amino acids when evolving in male-biased populations is consistent with the prediction of sexual conflict theory that males should evolve sexually antagonistic adaptations to manipulate females to increase their own fitness [2,3]. The increase in male fitness with spermatophylax mass is well documented in *G. sigillatus* and occurs because heavier spermatophylaxes take longer for the female to consume, facilitating greater sperm transfer [35,40]. This relationship is also well supported in a range of other insect species, irrespective of whether the nuptial gift being provided is endogenous (e.g. [33,62]) or exogenous (e.g. [63,64]) in origin. The effects of free amino acids in the nuptial gift on male fitness, however, are not as well documented. In the German cockroach (*Blattella germanica*), amino acids in the male tergal gland secretions act as phagostimulants to entice mating [65]. Free amino acids in the spermatophylax of *G. sigillatus* also act as phagostimulants, even in the non-gift giving cricket *Acheta domesticus* [46,47]. More importantly, free amino acids in a specific combination (used here to construct a multivariate manipulation score) reduce the likelihood that this nuptial gift will be prematurely discarded before complete sperm transfer has occurred, most likely by altering the texture and/or gustatory appeal of the spermatophylax [42]. This pattern of chemical manipulation differs from that observed in *D. melanogaster* [66], as well as in many other insect species [27], where manipulative chemicals (such as seminal fluid and accessory gland proteins) are transferred in the male ejaculate as a genital gift. While sex peptides are predicted to be more common in endogenous transdermal and genital gifts [24], the spermatophylax in *G. sigillatus* also contains 30 novel proteins, over half of which arise from genes expressed in the male accessory glands [43]. Moreover, consumption of the spermatophylax elicits distinct gene expression patterns in the head of females [67] and reduces spermatophylax feeding duration in subsequent matings [58]. However, it is not currently known whether these spermatophylax proteins have also evolved in response to interlocus sexual conflict. Collectively, our work adds to the growing list of insect studies showing that males evolve to become more manipulative under an increased intensity of interlocus sexual conflict (e.g. [39,68–70]), but also highlights how nuptial gifts can evolve to become more manipulative in a number of different ways.

Sexual conflict theory predicts that as males evolve sexually antagonistic adaptations to manipulate females, females should respond by evolving counter-adaptations to prevent this manipulation [2,3]. Indeed, females in a range of insect species have evolved increased resistance to male manipulation, with classic examples including the evolution of abdominal spines to prevent costly pre-mating struggles in water striders [71], thicker copulatory ducts to mitigate the damage caused by spiny genitalia in seed beetles [6] and the reduced sensitivity to the harmful effects of seminal fluid proteins in *D. melanogaster* [39,68]. In agreement with these studies, we show that both the regime of interlocus sexual conflict experienced by a female and the origin of the spermatophylax had a significant effect on female spermatophylax feeding behaviour in *G. sigillatus*. When receiving a spermatophylax from their own population, females evolving in male-biased populations spent longer feeding on this gift compared with those evolving in female-biased populations, but this pattern was reversed when receiving the gift from the opposing selection regime. This pattern of female feeding behaviour is not altogether surprising as it largely confirms that females feed for longer on a spermatophylax that is more manipulative. However, the most informative comparison is between the spermatophylax feeding times of females from male- and female-biased populations when they receive a gift that originates from the same selection regime. When the spermatophylax originated from the same selection regime, females evolving in male-biased populations always had shorter feeding durations than those evolving in female-biased populations indicating the evolution of greater resistance in these females. While females do not gain any obvious nutritional benefits by consuming the spermatophylax in *G. sigillatus* [54,56], the ability to resist feeding on this nuptial gift enables females to express post-copulatory mate choice [57] and provides a higher potential for polyandry which is known to provide genetic benefits to offspring in this species [56]. Female resistance will also have important consequences for males by reducing sperm transfer [35]. Based on the sperm transfer rates given in Sakaluk [35] for *G. sigillatus*, the reduced spermatophylax feeding durations observed in females evolving in male-biased populations is predicted to result in a 25.31% and 24.27% reduction in the number of sperm transferred when the gift was received from a male-biased and a female-biased population, respectively. Given that females are highly polyandrous and the outcome of sperm competition conforms to a numerical lottery in *G. sigillatus* [48–50], it is likely that the observed differences in spermatophylax feeding times and predicted rates of sperm transfer will also have important consequences for male paternity in this species.

Sexually antagonistic coevolution arises when evolutionary change in one sex modifies the selection acting on the opposite sex so that the sexes continually drive evolutionary change in each other [72]. While only one possible outcome of interlocus sexual conflict, there is extensive theoretical support for escalating ‘arms races’ driving the rapid diversification of the male and female traits mediating the conflict (e.g. [73,74]), facilitating population divergence (e.g. [73,75]) and even promoting speciation under certain conditions (e.g. [10,12,13]). However, sexually antagonistic coevolution is not well characterized for many species, as it requires a detailed understanding of the sexually antagonistic traits in both sexes [72]. Notable exceptions to this pattern include water striders (e.g. [5,71]), plant bugs (e.g. [76]), seed beetles (e.g. [6,69]), diving beetles (e.g. [77,78]), dung flies (e.g. [7]) and *D. melanogaster* (e.g. [39,68]), where a combination of comparative and experimental evolution approaches have been used to provide support for the operation of sexually antagonistic coevolution. In *G. sigillatus*, there is a positive genetic correlation between female spermatophylax feeding duration and the manipulative combination of free amino acids present in this gift [59], which demonstrates the clear potential for these traits to coevolve in the sexes. In this study, we provide direct evidence for the coevolution of these traits across the sexes, as well as between female spermatophylax feeding duration and the weight of the spermatophylax. Across experimental populations, we show that both manipulative properties of the spermatophylax we examined were positively correlated with female spermatophylax feeding duration, indicating the coevolution of these traits in response to interlocus sexual conflict. While our work shows the sexually antagonistic coevolution of manipulative and resistance traits in as little as 25 generations, it raises the obvious question as to whether these observed changes have also resulted in the evolution of reproductive isolation in these *G. sigillatus* populations. In general, the potential for interlocus sexual conflict to drive reproductive isolation has yielded mixed results, with some empirical studies on insects providing support for this association (e.g. [7,8]) but not others (e.g. [79,80]). Clearly, more work is needed on these experimental populations before the broader evolutionary implications of our findings on the mating system of *G. sigillatus* will be fully understood.

Experimental evolution is a powerful tool for understanding how traits evolve, but, in many cases, the mechanism(s) driving this evolutionary change are not known. While we believe that interlocus sexual conflict is the most parsimonious explanation for our results, we must acknowledge several alternate explanations. First, as we did not rear our crickets for two generations in a common garden setting (i.e. 50–50% adult sex ratio), it is possible that the adult sex ratio experienced by grandparents has influenced our final measurements of male spermatophylax properties and female feeding behaviour. However, while increased exposure to sexual interactions in parents can influence traits in offspring (e.g. [81]), we know of no study that has explicitly examined the transgenerational effects of adult sex ratio or whether these effects can extend across two generations. Second, differences in the opportunity for males to mate across our selection regimes will reduce the effective population size (Ne) in our male-biased populations. It is possible that this will increase inbreeding and reduce the genetic variance in our male-biased populations that influence our measurements of male spermatophylax properties and female feeding behaviour. However, we believe this is unlikely to be the case as we did not observe reduced fitness (e.g. reduced offspring production or increased mortality) in any of our populations during the 25 generations of our study. Moreover, if a lower Ne in male-biased populations increased inbreeding, we would expect to observe a reduction in fitness-related traits owing to inbreeding depression. The larger and more manipulative combination of free amino acids in the spermatophylax and shorter spermatophylax feeding times of females (i.e. greater resistance) suggests this is not the case. Finally, as the intensity of sexual selection will also vary across our selection regimes, it is possible that sexual selection may explain the observed differences in spermatophylax properties and female feeding responses even in the absence of sexual conflict. For example, the larger spermatophylaxes observed in male-biased populations may have evolved to provide information on the genetic quality of the male. However, this does not explain the more manipulative combination of free amino acids in the spermatophylax or reduced female feeding behaviour in these populations, nor the general finding that females do not receive any direct or indirect benefits from spermatophylax consumption in this species [54–56]. An increase in sperm competition in male-biased populations could explain these patterns in both spermatophylax properties, as they will facilitate a greater transfer of sperm. However, it does not explain the reduced female spermatophylax feeding times observed in male-biased populations unless females alter their feeding behaviour to bias the outcome of sperm competition. Indeed, this divergence in the interests of the sexes, we argue, generates interlocus sexual conflict in this species—males produce a manipulative spermatophylax to increase their own paternity, and females attempt to resist this manipulation to bias paternity towards their male(s) of choice. More generally, this highlights how sexual selection is a ready source of sexual conflict [82] and that these two processes should not be presented as alternative explanations for trait evolution.

## Data Availability

The data that support the findings of this article are available on Dryad [83]. Supplementary material is available online [84].
